# Supplementary material for: Serotonin modulates asymmetric learning from reward and punishment in healthy human volunteers
Source: Commun Biol. 2022 Aug 12;5:812. doi: 10.1038/s42003-022-03690-5 (PMC9374781; doi:10.1038/s42003-022-03690-5)
Supplement: Supplementary file 2 — Supplementary Information [file 42003_2022_3690_MOESM2_ESM.pdf]

Serotonin modulates asymmetric learning from reward and punishment in healthy human volunteers

Supplementary Material

Model number	1	2	3	4	5	6
iBIC session I	16446	10487	8419	7730	8045	7689
iBIC session II	16465	10934	8585	7440	8025	7428
iBIC session I and II	32911	21421	17004	15170	16070	15117

Supplementary Table 1. *Model comparison.*

We compared different models in terms of how well they explained subjects' choices on each session. For each model, iBIC scores (integrated Bayesian Information Criterion) are shown. A lower iBIC score indicates better fit with subjects' choices. The best-fitting model 6 is indicated in red.

Model 1 ('*gambling bias*'); Model 2 ('*gambling bias & computer number*'); Model 3 ('*Q-learning*'); Model 4 ('*adjusted Q-learning*'); Model 5 ('*asymmetric Q-learning*'); Model 6 ('*adjusted & asymmetric Q-learning*'). Cf. Methods for details.

Additional models tested, inferior fit across both sessions: a model with three learning rates, one for each deck type (iBIC session I and II: 16394); a model with asymmetric sensitivity to outcomes (win/loss) instead of asymmetry in learning rates (iBIC session I and II: 15126). A model with asymmetric sensitivity to outcomes and asymmetry in learning rates (iBIC session I and II: 15148).

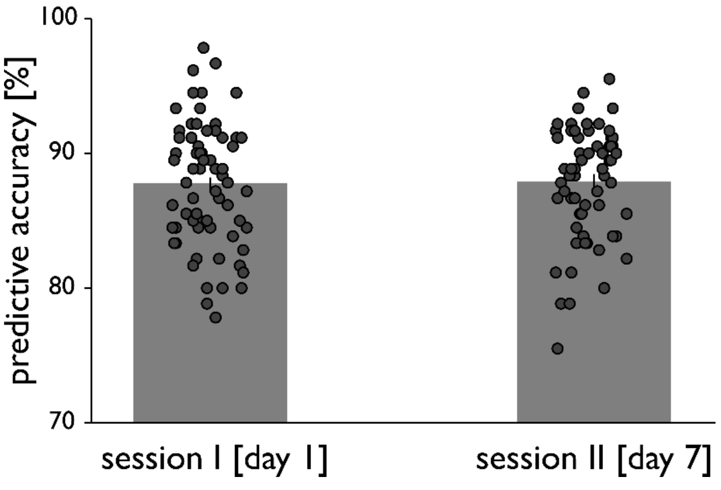

Supplementary Figure 1. *Predictive accuracy.*

The predictive accuracy of the model (absolute fit), i.e., the proportion of subjects' choices to which the model gives a likelihood greater than 50% (percent correct), was, on average, 87.71% for session I, and 87.92% for session II. Error bars indicate SEM.

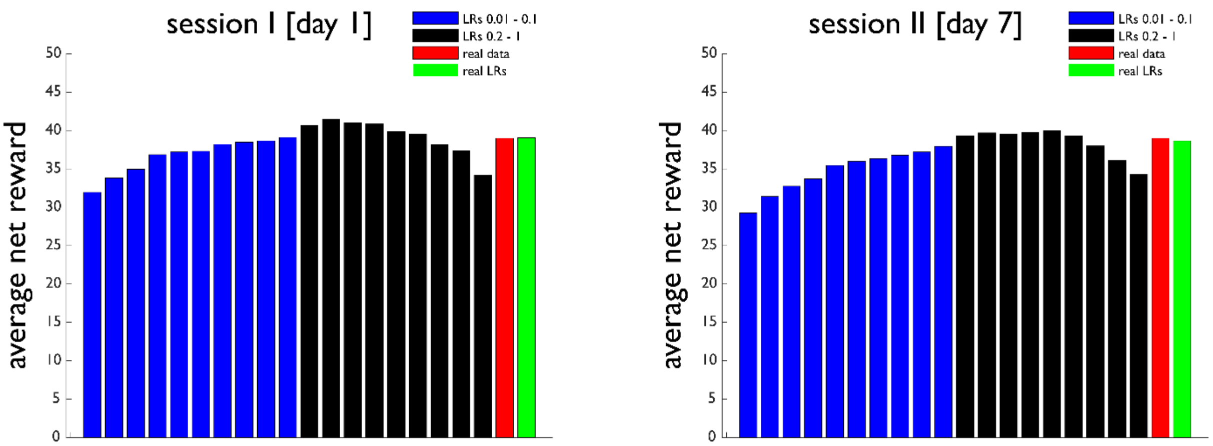

Supplementary Figure 2. *Simulations with different learning rates (LRs).*

We simulated data for artificial agents (n=66, 10 data sets each) with different LRs, ranging from 0.01-0.09 (blue) and 0.1-1 (black), respectively and computed the average net reward gained in the experiment. We also compared this to the net reward gained using LRs derived from fitting to real data (green), as well as to the net reward gained by subjects in the real experiment (red). This analysis revealed that LRs in the range of ≈0.1-0.7 reap highest reward, with an 'optimal' LR in the range of ≈0.3-0.6. The results also indicate that lower (e.g., ≤0.05), and higher (≥0.8) LRs are detrimental to performance. Overall, the mean LR at the population level in our data was 0.125, which allowed subjects, in the real experiment, to reap a net reward that was close to the net reward of artificial agents with an 'optimal' LR.

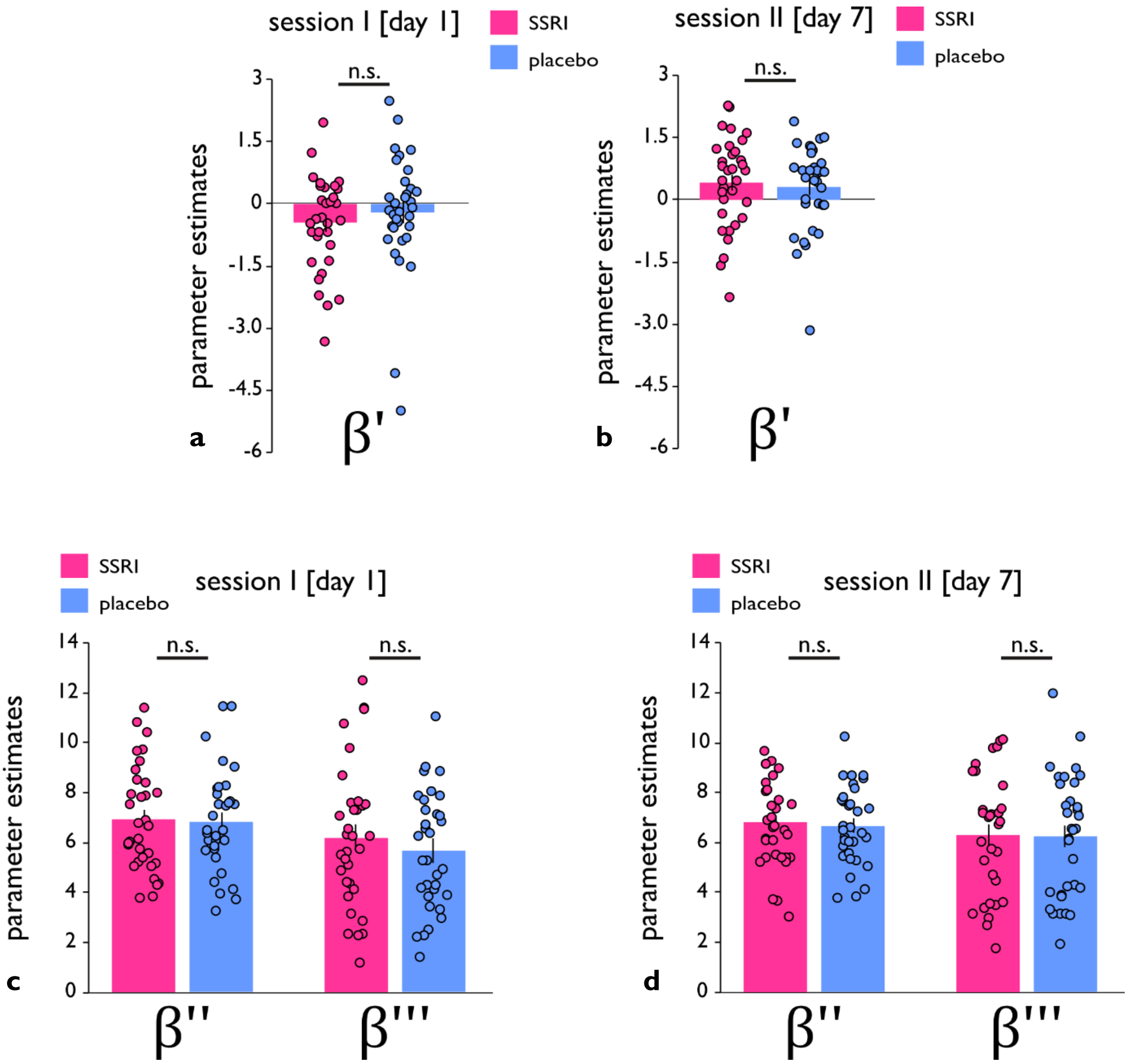

Supplementary Figure 3. *No drug effects on other model parameters.*

In contrast to the effect of SSRI treatment on learning rates, there were no between-group differences for the remaining model parameters, such as **(a&b)** the gambling bias parameter (β'; session 1: p=0.87, session 2: p=0.78), and **(c&d)** the decision temperature parameter determining the impact of the computer number (β''; session1: p=0.40, session 2: p=0.97) and the impact of learned Q-values (β''' session1: p=0.44, session 2: p=0.64). n.s. = not significant (p>0.05). Error bars indicate SEM.

Note that a gambling bias (β') was overall negatively associated with a learning asymmetry (r=-0.18, P=0.03), in line with the notion that subjects with a positive gambling bias (more likely to gamble to begin with) have more room to adjust their behaviour through learning from negative events (negative learning asymmetry, $\eta^- > \eta^+$).

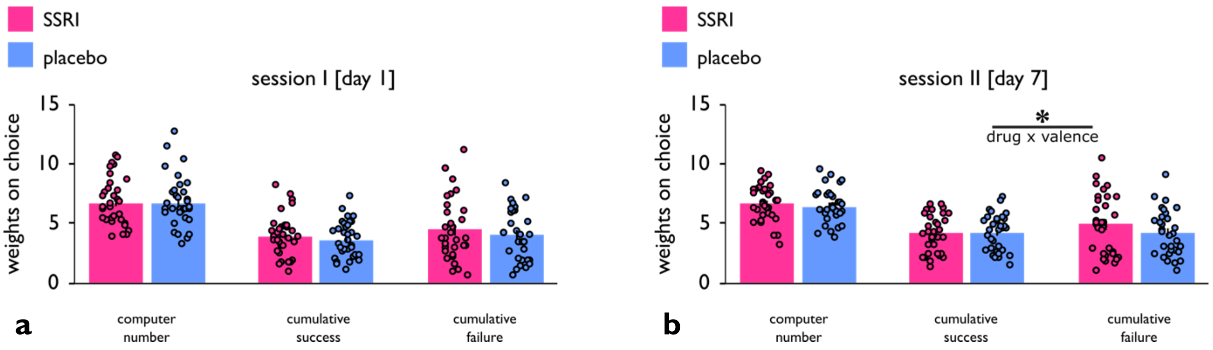

Supplementary Figure 4. *Results of trial-by-trial logistic regression model for simulated data (posterior predictive check).*

Generating simulated data based upon the model parameter estimates from the best-fitting model showed that the model captured core features of the real data. Fitting a logistic regression model to subjects' simulated decisions revealed highly similar effects of computer number, as well as cumulative success and failure for **(a)** session I, and **(b)** session II, respectively (cf. Fig. 2 of the main manuscript for the results of the real data). On session I, impact of cumulative success and failure was unaffected by treatment. On session II, however, SSRIs induced an asymmetric effect on reward and punishment (drug x valence: $F_{1,64}$=4.6, p=0.035). Note that for this analysis, we simulated 100 data sets and averaged the results. *p<0.05. Error bars indicate SEM.

Additionally, there was a strong positive correlation (all p<0.001) between regression weights for real and simulated data: Session I: computer number (r=0.891); cumulative success (r=0.816); cumulative failure (r=0.838). Session II: computer number (r=0.928); cumulative success (r=0.864); cumulative failure (r=0.899).

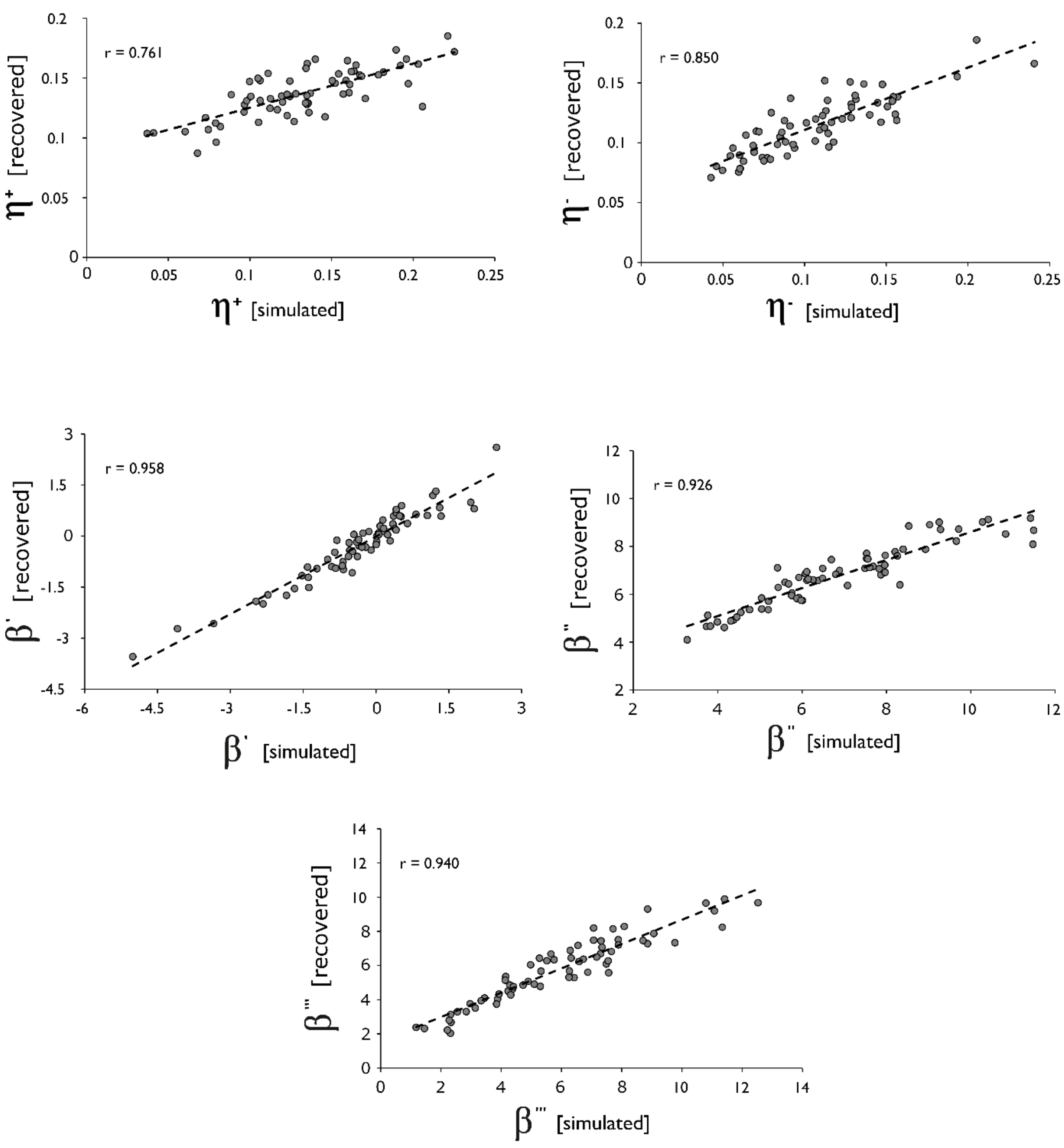

Supplementary Figure 5. *Recovery of model parameter estimates, session I.*

Model parameter estimates could be accurately recovered for session I. This is indicated by a strong positive correlation (all $p<0.001$) between the original parameters used for data simulation ('simulated') and the fitted parameters ('recovered'). β' = gambling bias parameter; β'' = decision temperature parameter determining the impact of the computer number; β'''= decision temperature parameter determining the impact of learned Q-values; η^+ = learning rate for reward; η^- = learning rate for punishment.

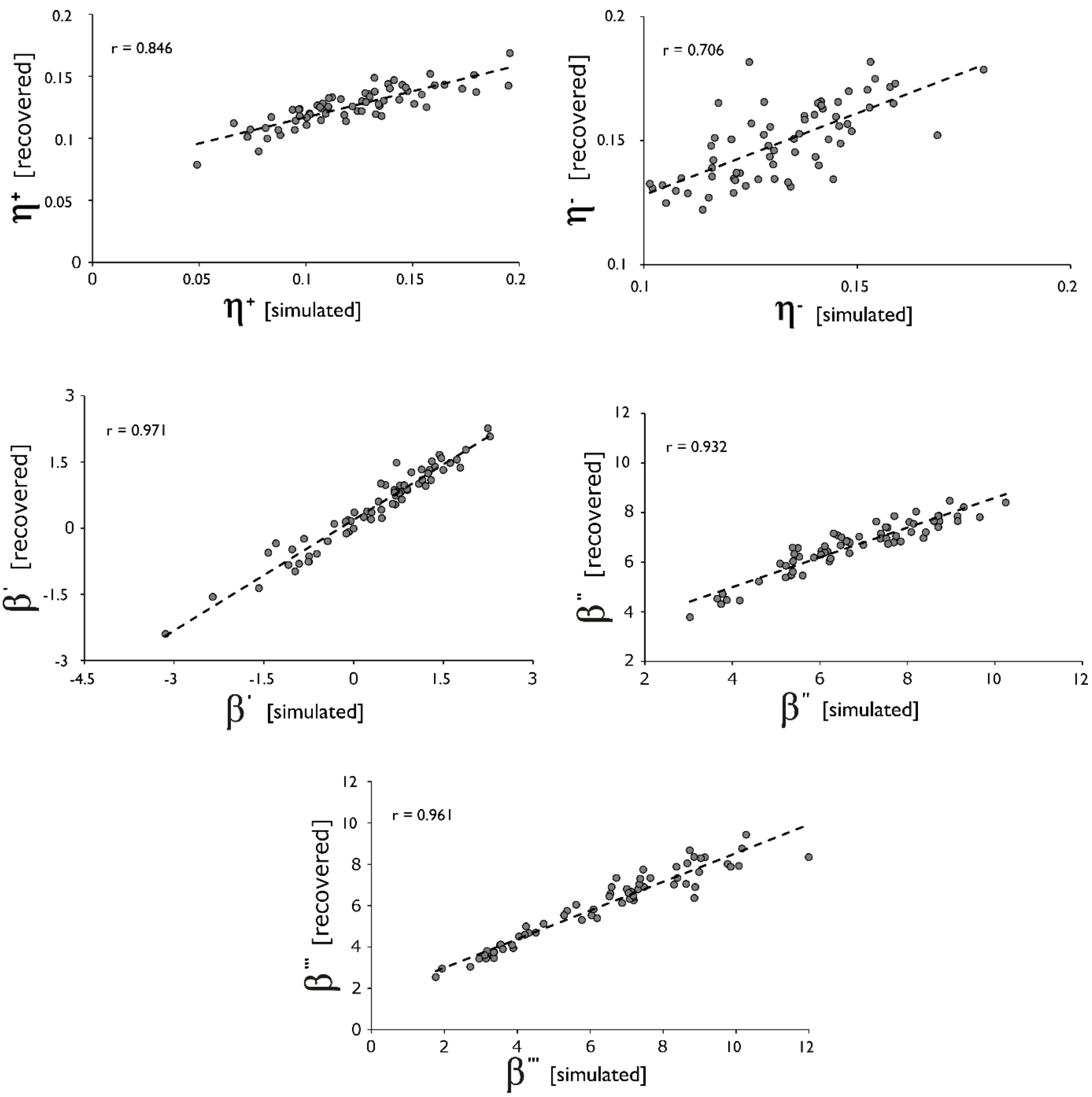

Supplementary Figure 6. *Recovery of model parameter estimates, session II.*

Model parameter estimates could be accurately recovered for session II. This is indicated by a strong positive correlation (all $p<0.001$) between the original parameters used for data simulation ('simulated') and the fitted parameters ('recovered'). β' = gambling bias parameter; β'' = decision temperature parameter determining the impact of the computer number; β'''= decision temperature parameter determining the impact of learned Q-values; η^+ = learning rate for reward; η^- = learning rate for punishment.

detected model

simulated model	1	2	3	4	5	6
1	10	0	0	0	0	0
2	0	10	0	0	0	0
3	0	0	10	0	0	0
4	0	0	0	10	0	0
5	0	0	0	0	10	0
6	0	0	0	0 [3]	0	7 [3]

Supplementary Figure 7. *Validation of the model comparison procedure*.

We simulated 10 data sets across both sessions, using each model with its parameters fitted to subjects' real choices, and we applied the model comparison procedure to each data set. Each cell shows how many datasets generated by the model indicated on the vertical axis were detected as reflecting the model used for simulation indicated on the horizontal axis. This analysis showed that the model comparison could accurately detect the model used for data simulation as the best-fitting model when using models 1-5 (10 out of 10 times, indicated in red), confirming specificity of the model comparison procedure, i.e., model 6 is not recognized when it is not the true underlying model. Moreover, this procedure confirmed sensitivity of the model comparison model procedure, i.e., model 6 is recognized when it is the true underlying model (10 out of 10 times). This was the case 7/10 times as the sole winner, i.e., iBIC difference > 6 to second best model, and 3/10 times as shared winner, i.e., within 6 of the lowest iBIC.

	Placebo		SSRI		P_{value}
Gender	20 ♀ / 13 ♂		20 ♀ / 13 ♂		1.000
Age	24.8 ± 3.9		24.5 ± 4.0		0.757
BDI – II [day 1]	4.4 ± 5.4		3.6 ± 4.0		0.540
BDI – II [day 7]	4.6 ± 5.7		4.5 ± 4.5		0.924
BDI – II [day 7 – day 1]	0.2 ± 3.3		0.8 ± 3.5		0.469
SHAPS [day 1]	0.3 ± 1.0		0.3 ± 0.7		1.000
SHAPS [day 7]	0.6 ± 1.6		0.8 ± 2.5		0.771
SHAPS [day 7 – day 1]	0.3 ± 1.5		0.5 ± 2.1		0.738
STAI - state [day 1]	30.6 ± 8.5		30.1 ± 6.4		0.795
STAI - state [day 7]	33.1 ± 9.7		31.4 ± 6.6		0.392
STAI - state [day 7 – day 1]	2.5 ± 8.5		1.4 ± 5.6		0.508
STAI - trait [day 1]	33.1 ± 9.7		34.6 ± 6.6		0.479
STAI - trait [day 7]	34.6 ± 9.8		35.5 ± 7.5		0.664
STAI - trait [day 7 – day 1]	1.5 ± 5.0		0.9 ± 3.1		0.615
PANAS - positive [day 1]	31.2 ± 8.6		30.0 ± 7.9		0.562
PANAS - positive [day 7]	29.0 ± 10.4		28.3 ± 8.3		0.775
PANAS - positive [day 7 – day 1]	-2.3 ± 7.3		-1.7 ± 5.6		0.749
PANAS - negative [day 1]	11.5 ± 2.5		11.2 ± 1.4		0.588
PANAS - negative [day 7]	12.1 ± 3.3		11.1 ± 1.7		0.143
PANAS - negative [day 7 – day 1]	0.5 ± 3.2		-0.2 ± 1.8		0.277

Supplementary Table 2. *Affective state questionnaire data.*
Drug groups were matched for age and gender, and there was no baseline difference in any of the affective state questionnaires (assessed on day 1, pre-drug). Moreover, there was no drug effect on any of the affective state measures. BDI – II = Beck's Depression Inventory II, SHAPS = Snaith-Hamilton Pleasure Scale, STAI = State-Trait Anxiety Inventory, PANAS = Positive and Negative Affective Scale.
