# Supplementary material for: Serotonin modulates asymmetric learning from reward and punishment in healthy human volunteers
Source: Commun Biol. 2022 Aug 12;5:812. doi: 10.1038/s42003-022-03690-5 (PMC9374781; doi:10.1038/s42003-022-03690-5)
Supplement: Supplementary file 5 — Reporting Summary [file 42003_2022_3690_MOESM5_ESM.pdf]

nature research

Corresponding author(s): Michely

Last updated by author(s): Apr 12, 2022

Reporting Summary

Nature Research wishes to improve the reproducibility of the work that we publish. This form provides structure for consistency and transparency in reporting. For further information on Nature Research policies, see our Editorial Policies and the Editorial Policy Checklist.

Statistics

For all statistical analyses, confirm that the following items are present in the figure legend, table legend, main text, or Methods section.

n/a	Confirmed	
☐	☒	The exact sample size (*n*) for each experimental group/condition, given as a discrete number and unit of measurement
☐	☒	A statement on whether measurements were taken from distinct samples or whether the same sample was measured repeatedly
☐	☒	The statistical test(s) used AND whether they are one- or two-sided<br>*Only common tests should be described solely by name; describe more complex techniques in the Methods section.*
☐	☒	A description of all covariates tested
☐	☒	A description of any assumptions or corrections, such as tests of normality and adjustment for multiple comparisons
☐	☒	A full description of the statistical parameters including central tendency (e.g. means) or other basic estimates (e.g. regression coefficient) AND variation (e.g. standard deviation) or associated estimates of uncertainty (e.g. confidence intervals)
☐	☒	For null hypothesis testing, the test statistic (e.g. *F*, *t*, *r*) with confidence intervals, effect sizes, degrees of freedom and *P* value noted<br>*Give P values as exact values whenever suitable.*
☒	☐	For Bayesian analysis, information on the choice of priors and Markov chain Monte Carlo settings
☒	☐	For hierarchical and complex designs, identification of the appropriate level for tests and full reporting of outcomes
☐	☒	Estimates of effect sizes (e.g. Cohen's *d*, Pearson's *r*), indicating how they were calculated

Our web collection on statistics for biologists contains articles on many of the points above.

Software and code

Policy information about availability of computer code

Data collection	MATLAB 2010b (Mathworks); Psychtoolbox for MATLAB (http://psychtoolbox.org/)
Data analysis	MATLAB 2017b (Mathworks); SPSS 24 (IBM)

For manuscripts utilizing custom algorithms or software that are central to the research but not yet described in published literature, software must be made available to editors and reviewers. We strongly encourage code deposition in a community repository (e.g. GitHub). See the Nature Research guidelines for submitting code & software for further information.

Data

Policy information about availability of data

All manuscripts must include a data availability statement. This statement should provide the following information, where applicable:

- Accession codes, unique identifiers, or web links for publicly available datasets
- A list of figures that have associated raw data
- A description of any restrictions on data availability

Data and code for reproducing the associated analyses are available on GitHub: https://github.com/jmichely/ssri_asymmetric_learning.

Field-specific reporting

Please select the one below that is the best fit for your research. If you are not sure, read the appropriate sections before making your selection.

☒ Life sciences ☐ Behavioural & social sciences ☐ Ecological, evolutionary & environmental sciences

For a reference copy of the document with all sections, see nature.com/documents/nr-reporting-summary-flat.pdf

Life sciences study design

All studies must disclose on these points even when the disclosure is negative.

Sample size	We selected a sample size that is based on related studies using a similar pharmacological protocol (e.g., Grillon et al., 2009, Neuropsychopharmacology; Scholl et al., 2017, Plos Biol; Skandali et al., 2018, Neuropsychopharmacology).
Data exclusions	The logistic regression analysis did not converge for one subject on session I, thus we discarded this data from the group analysis.
Replication	We did not attempt to reproduce the pharmacological results. However, note that the results of our study on learning asymmetries replicate previous findings (Eldar et al., 2016, PNAS).
Randomization	Subjects were randomly allocated into two equal sized groups (SSRI / placebo), controlling for an equal gender balance across both groups.
Blinding	The study followed a double-blind protocol.

Reporting for specific materials, systems and methods

We require information from authors about some types of materials, experimental systems and methods used in many studies. Here, indicate whether each material, system or method listed is relevant to your study. If you are not sure if a list item applies to your research, read the appropriate section before selecting a response.

Materials & experimental systems

n/a	Involved in the study	
☒	☐	Antibodies
☒	☐	Eukaryotic cell lines
☒	☐	Palaeontology and archaeology
☒	☐	Animals and other organisms
☐	☒	Human research participants
☒	☐	Clinical data
☒	☐	Dual use research of concern

Methods

n/a	Involved in the study	
☒	☐	ChIP-seq
☒	☐	Flow cytometry
☒	☐	MRI-based neuroimaging

Human research participants

Policy information about studies involving human research participants

Population characteristics	Measured population characteristics included age, gender, and questionnaire data. Note, however, that none of these measures were used as covariates for any of the analyses.
Recruitment	Participants were recruited from a participant pool at University College London, via leaflets on University College London campus, and via an online community platform for researchers and research participants (https://www.callforparticipants.com).
Ethics oversight	University College London local research ethics committee (Ethics-ID: 9787/002, Serotonergic modulation of emotional processing, decision-making and learning).

Note that full information on the approval of the study protocol must also be provided in the manuscript.
